# Supplementary material for: Modelling of performance prediction by analysis of elite swimmers’ anthropometry, peak performance age and age-related performance progression
Source: PLoS One. 2025 Sep 17;20(9):e0332306. doi: 10.1371/journal.pone.0332306 (PMC12443251; doi:10.1371/journal.pone.0332306)
Supplement: S1 Table — (DOCX) [file pone.0332306.s001.docx]

**S1 Table**. Models introduced by dynamic panel data using top list results (supplementary for figure 4).

| **Styles; Events** | | | **Sex** | **Model**  **P-Value** | **Sargan test**  **p-value** |
| --- | --- | --- | --- | --- | --- |
| Free Style | 1 | 50m | M | R_t_ =0.4189178(R_t-1_-m.R_t-1_)+0.9982353(m.R_t_)  P<0.001; P= 2.138e-10, <2.2e-16 | 0.99567 |
|  |  |  | W | R_t_ =0.4059650(R_t-1_-m.R_t-1_)+0.9974553(m.R_t_)  P<0.001; P= 1.872e-08, <2.2e-16 | 0.99883 |
|  | 2 | 100m | M | R_t_ =0.527198(R_t-1_-m.R_t-1_)+0.998877(m.R_t_)  P<0.001; P= 6.22e-13, <2.2e-16 | 0.98626 |
|  |  |  | W | R=0.59971792(R_t-1_-m.R_t-1_)+0.99884913(m.R_t_)  P<0.001; P= <2.2e-16, <2.2e-16 | 0.99653 |
|  | 3 | 200m | M | R_t_ =0.46711488(R_t-1_-m.R_t-1_)+0.99982695(m.R_t_)  P<0.001; P=1.347e-11, <2.2e-16 | 0.96819 |
|  |  |  | W | R_t_ =0.4932889(R_t-1_-m.R_t-1_)+0.9979652(m.R_t_)  P<0.001; P= 7.425e-12, <2.2e-16 | 0.99604 |
|  | 4 | 400m | M | R_t_=0.5410500(R_t-1_-m.R_t-1_)+0.9987102(m.R_t_)  P<0.001; P= 2.596e-08, <2.2e-16 | 0.99416 |
|  |  |  | W | R_t_ =0.5663396(R_t-1_-m.R_t-1_)+0.9981898(m.R_t_)  P<0.001; P= 1.377e-07, <2.2e-16 | 0.9967 |
|  | 5 | 800m | M | R_t_=0.4313780(R_t-1_-m.R_t-1_)+1.0001137(m.R_t_)  P<0.001; P= 3.672e-05, <2.2e-16 | 0.98256 |
|  |  |  | W | R_t_ =0.5597385(R_t-1_-m.R_t-1_)+0.9983189(m.R_t_)  P<0.001; P=6.817e-05, <2.2e-16 | 0.95956 |
|  | 6 | 1500m | M | R_t_=0.5615849(R_t-1_-m.R_t-1_)+1.0004384(m.R_t_)  P=<0.001; P= 0.0001744, <2.2e-16 | 0.9975 |
|  |  |  | W | R_t_ =0.5155478(R_t-1_-m.R_t-1_)+0.9975740(m.R_t_)  P<0.001; P= 6.817e-05, <2.2e-16 | 0.94479 |
| Back Stroke | 7 | 50m | M | R_t_=0.4597754(R_t-1_-m.R_t-1_)+0.9974493(m.R_t_)  P<0.001; P= 6.604e-7, <2.2e-16 | 0.77873 |
|  |  |  | W | R_t_ =0.62875731(R_t-1_-m.R_t-1_)+1.00041353(m.R_t_)  P<0.001; P= 1.677e-14, <2.2e-16 | 0.99684 |
|  | 8 | 100m | M | R_t_=0.659334(R_t-1_-m.R_t-1_) +0.998212(m.R_t_)  P<0.001; P= <2.2e-16, <2.2e-16 | 0.99847 |
|  |  |  | W | R_t_ =0.2248114(R_t-2_-m.R_t-2_) +0.3132554(R_t-1_-m.R_t-1_)+ 0.9989237(m.R_t_)+0.0081828(W)  P<0.005,<0.001,<0.001, P= 0.002101, 9.23e-5, <2.2e-16, 0.008294 | 0.99515 |
|  | 9 | 200m | M | R_t_=0.5000296(R_t-1_-m.R_t-1_)+1.0001545(m.R_t_)  P<0.001; P= 9.44e-13, <2.2e-16 | 0.99869 |
|  |  |  | W | R_t_ =0.527796(R_t-1_-m.R_t-1_)+0.998620(m.R_t_)  P<0.001; P= 5.119e-08, <2.2e-16 | 0.96095 |
| Breaststroke | 10 | 50 m | M | R_t_ =0.3305586(R_t-2_-m.R_t-2_)+0.3687685(R_t-1_-m. R_t-1_)+0.9977772(m.R_t_)  P<0.001, <0.005; P= 1.198e-06, 0.002425, <2.2e-16 | 0.99766 |
|  |  |  | W | R_t_ =0.6678541(R_t-1_-m.R_t-1_)+0.9963266(m.R_t_)  P<0.001; P= <2.2e-16, <2.2e-16 | 0.89272 |
|  | 11 | 100 m | M | R_t_=0.66980577(R_t-1_-m.R_t-1_)+0.99837089(m.R_t_)  P<0.001; P= <2.2e-16, <2.2e-16 | 0.94468 |
|  |  |  | W | R_t_ =0.2268992(R_t-2_-m.R_t-2_)+0.3785203(R_t-1_-m.R_t-1_)+0.9962938(m.Rt)  P<0.005, <0.001; P= 0.00135, 8.174e-05, <2.2e-16 | 0.9638 |
|  | 12 | 200 m | M | R_t_=0.5326479(R_t-1_-m.R_t-1_)+0.9982567(m.R_t_)  P<0.001; P= 1.392e-08, <2.2e-16 | 0.99667 |
|  |  |  | W | R_t_ =0.2255104(R_t-2_-m.R_t-2_)+0.3944463(R_t-1_-m.R_t-1_)+0.9980299(m.R_t_)  P<0.001, <0.001; P= 4.543e-05, 1.526e-09, <2.2e-16 | 0.97397 |
| Butterfly | 13 | 50m | M | R_t_=0.4900990(R_t-1_-m.R_t-1_)+0.9977391(m.R_t_)  P<0.001; P= 2.37e-10, <2.2e-16 | 0.95783 |
|  |  |  | W | R_t_ =0.4219795(R_t-1_-m.R_t-1_)+0.9987497(m.R_t_)  P<0.01; P= 0.009096, <2.2e-16 | 0.99882 |
|  | 14 | 100m | M | R_t_=0.3778307(R_t-1_-m.R_t-1_)+0.9974634(m.R_t_)  P<0.001; P= 1.923e-06, <2.2e-16 | 0.93527 |
|  |  |  | W | R_t_ =0.4347987(R_t-1_-m.R_t-1_)+0.9984855(m.R_t_)  P<0.01; P= 0.005137, <2.2e-16 | 0.99878 |
|  | 15 | 200m | M | R_t_=0.5957700(R_t-1_-m.R_t-1_)+0.9997376(m.R_t_)  P<0.001; P= 1.776e-11, <2.2e-16 | 0.99915 |
|  |  |  | W | R_t_ =0.1905362(R_t-2_-m.R_t-2_)+0.1905362(R_t-1_-m.R_t-1_) +0.9989237(m. R_t_)  P=0.01, P<0.01, <0.001; P= 0.01071, 1.311e-08, <2.2e-16 | 0.99787 |
| Medley | 16 | 200m | M | R_t_=0.6377491(R_t-1_-m.R_t-1_)+1.0004452(m.R_t_)  P<0.001; P= <2.2e-16, <2.2e-16 | 0.99783 |
|  |  |  | W | R_t_ =0.2893992(R_t-1_-m.R_t-1_)+0.9979079(m.R_t_)  P<0.005; P= 0.004386, <2.2e-16 | 0.99514 |
|  | 17 | 400m | M | R_t_=0.5503884(R_t-1_-m.R_t-1_)+0.9995769(m.R_t_)  P<0.001; P= 9.5e-11, <2.2e-16 | 0.99763 |
|  |  |  | W | R_t_ =0.3929723(R_t-1_-m.R_t-1_)+0.9996889(m.R_t_)  P<0.01; P= 0.0071674 | 0.99973 |

Rt is the predicted record at a given age, Rt -1 is the best personal record one year ago, Rt -2 is the best personal record two years ago, m.Rt is the average (mean) of the all-time top list records at a given age (Table 2), H is the athlete's height at adulthood, and W is the athlete's weight at adulthood. For example, future performances for a 16-year-old male swimmer in the 100 m freestyle with a height of 191 cm and a best performance of 53.21 s at age 15 are estimated to be 51.72, 49.47, 49.40, 49.40, 48.88, 48.49, and 48.16 s at ages 16, 17, 18, 19, 20, 21, and 22, respectively. Rt -1 is the best personal record one year ago, Rt -2 is the best personal record two years ago, Rt.m is the average (mean) of the records of the all-time top lists at a given age (Table 1). The factors for each discipline were set to a statistical significance of P<0.05. The R software dynamic panel data P-values are indicated in each case.
