# Supplementary material for: Modelling of performance prediction by analysis of elite swimmers’ anthropometry, peak performance age and age-related performance progression
Source: PLoS One. 2025 Sep 17;20(9):e0332306. doi: 10.1371/journal.pone.0332306 (PMC12443251; doi:10.1371/journal.pone.0332306)
Supplement: Attachment — Submitted filename: Responses to the Reviewers.docx [file pone.0332306.s004.docx]

**PONE-D-25-06170**

**Modelling of performance prediction by analysis of elite swimmers’ anthropometry, peak performance age and age-related performance progression**

Dear staff editor,

The authors acknowledge the constructive and positive feedback provided by the reviewer. Please see below our point-by-point response to the concerns, along with the revised manuscript with changes highlighted. We hope that the manuscript now qualifies for acceptance.

Sincerely,

The authors.

**Reviewers' comments:**

Reviewer's Responses to Questions

**Comments to the Author**

1. Is the manuscript technically sound, and do the data support the conclusions?

The manuscript must describe a technically sound piece of scientific research with data that supports the conclusions. Experiments must have been conducted rigorously, with appropriate controls, replication, and sample sizes. The conclusions must be drawn appropriately based on the data presented.

Reviewer #1: Yes

2. Has the statistical analysis been performed appropriately and rigorously?

Reviewer #1: Yes

3. Have the authors made all data underlying the findings in their manuscript fully available?

The [PLOS Data policy](https://eur01.safelinks.protection.outlook.com/?url=http%3A%2F%2Ftrack.editorialmanager.com%2FCL0%2Fhttp%3A%252F%252Fwww.plosone.org%252Fstatic%252Fpolicies.action%2523sharing%2F1%2F010f0195fa8f0292-723d5673-77d2-41ee-b5db-0e66ea10ea78-000000%2FwqHSuuZDjle4vU4gAEFekuP9n0toKVxCnQ-Q2iC_5Ns%3D205&data=05%7C02%7Cali.gorzi.2%40ki.se%7Ce4caa19d45bc43fb9c1408dd72816add%7Cbff7eef1cf4b4f32be3da1dda043c05d%7C0%7C0%7C638792622369045859%7CUnknown%7CTWFpbGZsb3d8eyJFbXB0eU1hcGkiOnRydWUsIlYiOiIwLjAuMDAwMCIsIlAiOiJXaW4zMiIsIkFOIjoiTWFpbCIsIldUIjoyfQ%3D%3D%7C0%7C%7C%7C&sdata=SqsesPQCoa0HBPAJ6Ikjhy6nd6vHP%2Fhhew8azr4Uw%2Bg%3D&reserved=0) requires authors to make all data underlying the findings described in their manuscript fully available without restriction, with rare exception (please refer to the Data Availability Statement in the manuscript PDF file). The data should be provided as part of the manuscript or its supporting information, or deposited to a public repository. For example, in addition to summary statistics, the data points behind means, medians and variance measures should be available. If there are restrictions on publicly sharing data—e.g. participant privacy or use of data from a third party—those must be specified.

Reviewer #1: No We have added all data as supplementary 2.

4. Is the manuscript presented in an intelligible fashion and written in standard English?

PLOS ONE does not copyedit accepted manuscripts, so the language in submitted articles must be clear, correct, and unambiguous. Any typographical or grammatical errors should be corrected at revision, so please note any specific errors here.

Reviewer #1: Yes

5. Review Comments to the Author

Please use the space provided to explain your answers to the questions above. You may also include additional comments for the author, including concerns about dual publication, research ethics, or publication ethics. (Please upload your review as an attachment if it exceeds 20,000 characters)

Reviewer #1: A review of the manuscript titled:

Modelling of performance prediction by analysis of elite swimmers’
anthropometry, peak performance age and age-related performance progression

The manuscript presents an interesting study on the developmental progression of sports performance of world-class swimmers. The study is enriched by a comparison of basic somatic indicators of two generations of swimmers, which should be better explained.
In this manuscript, interesting conjectures are briefly discussed, without an attempt to deepen the literature, which makes these considerations shallow, burdened with subjectivity, although often accurate.

Precise, specific suggestions for organizing the content and necessary changes are below.
Specific comments:

Abstract
What did the authors have in mind when writing the sentence in lines 17-19? Please change, explain.

Thank you for this comment. We agree that it was unclear. Revised; Please see the revised version.

Introduction
Lane 43 There are more current considerations and studies on the development of young swimming talents in the literature, and this is an important issue. Therefore, omitting important references, with one Spanish one, is a simplification.

Thank you for this comment. We have added further references.

Lines 46-50 Reference to publications and specific tests - water and general - land in the prediction of swimming efficiency and development would be justified, the reviewer notes the omission of relevant literature considerations that exist.

Revised. Please see the revised version.

Lines 51-53 Similarly, only one citation was used for authors from 2022. For example, a year earlier, one publication precisely on the topic of mediation between maturation, body physicality, and performance in female swimmers was published in the same journal, in others as well, later this year as well, which concerns swimming, not perception through other sports as in the manuscript.

Thank you for pointing this out. Revised.

Line 72, The insert regarding the aging aspect of swimming, is unnecessary without further involvement in this study.

An important aspect of the paper is “age and the age of peak performance”. We noted, in the results and discussion, that more recent men and women swimmers reach their peak performance earlier than former swimmers (fig 3). Thus, discussing the age and aging factor is relevant in the manuscript.

Lines 76-79 the authors should have introduced at least some information regarding somatic changes of the new generation vs. the previous one instead of mentioning aging of swimmers, because aging itself is not the subject of considerations in this work.

Thank you for this very relevant comment. We have made revisions in both the introduction and the discussion.

2. Materials and Methods and Results

Lines 95-96 PPA, height and weight analysis was performed on the same subjects as the swimming results, or was some other historical analysis of a similar group used? Please explain.

This has been clarified in the revised version: (these 20 …).

Lines 128 - 222 This data should be inserted in an orderly manner into tables, and the progression of results as curves on charts. However, the attached charts should be of higher quality, because they are simply illegible.

All data are presented in a table (Suppl. 1 for models, and table 1 for progression of results). The figure resolutions improves if you click on the link to open the actual figure files.

4. Discussion

Lines 285 - 287 please explain what is important?

Thank you for this comment. Revised.

In lines 288-297 the focus should be on the results rather than considering the effectiveness of the prediction model used.

We agree, thank you for this comment. See revised version.

Lines 309 – 310 “The 100m BK female swimmers were the only 100m swimmers who were heavier than their 50m counterparts in all men’s and women’s swimming strokes.” - Please clarify.

We agree that this was unclear. Revised.

Lines 322 – 323 References to biological age, puberty in girl swimmers and impact on athletic performance should be implemented.

Revised.

Line 324 “free style” > freestyle

Revised.

Similarly in line 337, without references to contemporary literature related to muscle mass and body composition of young swimmers.

Revised.

Only somewhere around line 350-4 should considerations regarding the perfection of the prediction model be included to structure the discussion, reflecting on increasing anaerobic capacity, experience, perhaps pace distribution, etc.?

Revised.

Line 358 (…) in multiple disciplines or in different swimming stroke races

Revised.
Line 359 “history of swimmers (training age)“ > training experience, years of experience

Revised.

Lines 361 - 363, if there is a mention of an age difference resulting from differences in the day, week, month in a given year, there should be a mention and a more current swimming reference to the differences in biological age.
Revised.

References

References should be supplemented according to the suggestions for the appropriate sections of the manuscript.

Revised.
