# Supplementary material for: Modelling of performance prediction by analysis of elite swimmers’ anthropometry, peak performance age and age-related performance progression
Source: PLoS One. 2025 Sep 17;20(9):e0332306. doi: 10.1371/journal.pone.0332306 (PMC12443251; doi:10.1371/journal.pone.0332306)
Supplement: Attachment — Submitted filename: Responses to Reviewers 2.docx [file pone.0332306.s005.docx]

**PONE-D-25-06170**

**Modelling of performance prediction by analysis of elite swimmers’ anthropometry, peak performance age and age-related performance progression**

Dear staff editor,

The authors acknowledge the constructive and positive feedback provided by the reviewer. Please see below our point-by-point response to the concerns, along with the revised manuscript with changes highlighted. We hope that the manuscript now qualifies for acceptance.

Sincerely,

The authors.

**Reviewers' comments:**

Reviewer #3: This is a sound paper and with some further attention, could be a welcome addition to peer-reviewed evidence on the topic.

Thank you very much.

ABSTRACT
Line 35: “Both male and female more recent swimmers were shorter, lighter and, in particular, younger than their former counterparts in most events.” Without preceding content outlining the nature of the comparison, this makes little sense as presented. I.e. you do not allude to a “more recent” vs “less recent” (I suppose) comparison being an aim/part of methods.

Thank you for this comment. Revised.

INTRODUCTION
Well written, nothing to add.

Thank you very much.

METHODS
Mostly minor issues for consideration.
Line 118-119: Can you explain in a little more detail how you determined whether a swimmer was deemed former or recent?

Thank you for this comment. Revised.

Line 122-123: double semi-colon and no closing bracket.

Revised. Please see the revised version.

Line 124: grammar… missing a “were included”?

Revised. Please see the revised version.

Line 131-132: A little more detail is needed when you refer to data being “excluded by boxplot”… be more specific if you can.

Thank you for this comment. Revised.

Line 145-148: Can you add a citation to support your division of training and test set according to the 90:10 split you have adopted? Only because 80:20 would be more conventional.

Since the test samples were not included in the modelling and the data was limited, based on the results of testing 90:10 and 80:20 in some disciplines as a pilot, we decided to prioritise 90:10 for the final presentation.

We have revised the manuscript to address this valuable comment.

Stat analysis:

1. can you clarify the model used with the panel data models (i.e. fixed effects, random effects, other?).

2. Were any assumptions checked (e.g. autocorrelation)?

3. Re. the 18 observations, can you add how these were identified…randomly I guess?

4. What statistics were used to evaluate the model fit (e.g. RMSE, AIC/BIC etc.).

5. With so many independent samples t-tests executed, the authors are encouraged to consider protection against type I errors.
6. Table 1 suggests some missing data (i.e. gaps), how were these dealt with (statistically) exactly?

Thank you for this precise comment. Revised. Please see the revised version.

1. In the revised manuscript, we confirmed that the model used is a panel data model with fixed effects.

2. To assess the validity of the model assumptions, we used the Arellano-Bond test to examine the absence of autocorrelation in the error terms. The related p-values were not included in the manuscript.

3. As you correctly guessed, the 18 observations in the set were randomly selected from the available data (highlighted in stats analysis).

4. Regarding model evaluation, instead of using the typical metrics such as RMSE, AIC, or BIC, which are not typically applicable in GMM estimation, we used the Sargan test to assess the validity of the instruments and the overall model fit.

5. We note that we did not perform simultaneous inference from the t-tests. Rather, each test was conducted in an exploratory context and the results were interpreted individually, without drawing collective conclusions from multiple comparisons. We have revised this and used the 'every event' approach instead of the 'all events' approach in the statistical analysis and results.

6. As noted in the table caption (M ± SD), Table 1 presents descriptive data, and we don’t have access to the missing data (basically in starting ages, before 13 years old, or in finishing ages, after 32 years old).

RESULTS
Line 153 and others: I feel presentation of your confidence intervals needs attention. E.g. “CI=.0040 .0566” would be better presented as “0.0040 to 0.0566”… repeat throughout results were applicable.

Revised throughout the results. Please see the revised version.

Line 152: you report an effect size here but there has been no mention of their use in the methods – can you go back and provide the details? I.e. are these partial eta-type ES stats or Cohen’s d? details about what is deemed small-large etc need adding too.

Thank you for this comment. Revised.

Line 154-155: “…and approaching significance for BF swimmers (P=0.53).”… this p-value is not approaching significance…missing zero? Regardless, it is not significant hence my suggestion is to remove this point (and any others about near significance like lines 164-169, 190-195). The results are already rather long, with many differences to comprehend, so this would aid interpretation for the reader.

Revised (0.053). Please see the revised version.

Line 195: Linked to the above I guess, “were insignificantly heavier” in essence means they were not (statistically) heavier!

Revised. Please see the revised version.

Lines 227-232: Suggest removal of all this content. Avoids repetition (i.e. we already know female differences from previous few lines), the details are cumbersome and many link to above comment re. non-sig. findings.

Revised. Please see the revised version.

Performance progression modelling: Can you provide an indication of the model fit for each presented equation? Perhaps add them to the figures themselves.

We included the Sargan p-values (model fit) in the supplementary table (S1) to avoid confusion in the figures.

DISCUSSION
First paragraph is more akin to a results subsection. Suggest re-write whereby you synthesise the key findings (i.e. that certain factors were of significance or not) rather than essentially listing every finding as you have.

Revised. Thank you for this very good comment.

Line 323: When stating 13/17 & 16/17 being lighter, this is based upon averages not the statistical findings hence it is exaggerated. Suggest re-writing with the numerator based on sig diffs only.

Revised. Please see the revised version.

Line 341: Replace “It seems” with “It is plausible”.

Revised. Please see the revised version.

Lines 373-374: This statement contradicts your panel data which regularly cited rt-1 and rt-2 (less often) as significant factors. Perhaps I have missed something here, but regardless, the point you are making needs attention so readers are not left confused. Indeed, on lines 401-403 you state “last year’s records in all events and last 2 years’ records in men’s 50m BT and women’s 100m BK, 100m BT, 200m BT and 200m BF, and weight in 100m BK, are important for predicting performance”.

Here, we are emphasising the worth of two years' best records versus one year.

However, we provided more information to better clarify and address your comment.

General: for all the work that has gone into the production of the panel analysis, you have not overly discussed these findings. I.e. the balance of attention is on your comparisons (recent/former, male/female, t-test comparing events etc) with less emphasis on these plots, what they demonstrate and how that might aid practitioners. Can you do so?

Basically, it’s difficult to discuss the modelling in more detail due to the limited literature available. Although all the discussed factors (height, weight and PPA) are key to our modelling, we have added some sentences more and have replaced some paragraphs and sentences to prioritise discussions related to modelling in response to your valuable comment. We have also highlighted the relevant discussions in yellow to show (only to reviewer) that our focus is on modelling.
